# The troubles with peer review for allocating research funding: Funders need to experiment with versions of peer review and decision‐making

**DOI:** 10.15252/embr.201949472

**Published:** 2019-11-03

**Authors:** Sandra Bendiscioli

**Affiliations:** ^1^ Science Policy EMBO Heidelberg Germany

**Keywords:** S&S: Ethics, S&S: Careers & Training

## Abstract

Peer review to allocate funding for researchers and projects has faced difficulties lately and come under criticism. Various alternatives and improvements are being tested to address these problems.

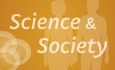

Peer review has been the most important mechanism for quality control in science for decades. In its basic form, it involves a group of scientific experts who judge the work of other scientists in order to allocate research funding, select scientific papers for publication, award recognitions, evaluate job applications and promotions, select presentations for conferences, and, more recently, investigate cases of scientific misconduct. As such, the academic enterprise's facility for generating knowledge and technologies depends in no small part on peer review to decide which ideas and projects should be supported. Overall, the peer‐review system is widely accepted and understood by the majority of researchers and trusted by policy makers. Given its efficiency and the widespread trust it enjoys, the Royal Society dubbed it as “the only effective way of properly assessing the quality of research proposals”.

the academic enterprise's facility for generating knowledge and technologies depends in no small part on peer review to decide which ideas and projects should be supported.

However, peer review has also been accompanied by criticism since it became the method of choice to assess the quality of science. Is it really an appropriate system for selecting the best people and the best ideas? Can reviewers be really objective when they have to judge their peers? In particular, as more researchers compete for grants, fellowships and academic positions, the task of reviewers is becoming increasingly difficult and time‐consuming, and some are concerned that that the peer‐review system is becoming overburdened.

What is referred to as “peer review” for allocating funding is actually a range of different approaches to collect peers’ expertise. The US National Science Foundation (NSF) for instance uses three variations: *ad‐hoc*‐only, panel‐only and *ad‐hoc* plus panel review. The *ad‐hoc*‐only review collects written comments from individual reviewers using the NSF's electronic system; the panel‐only method is based on in‐person discussions among reviewers; and many NSF grant schemes collect reviews through a combination of both methods (https://www.nsf.gov/nsb/publications/2016/nsb201641.pdf). Some funding agencies, such as the European Research Council (ERC) or Cancer Research UK (CRUK), use a two‐stage review process, in which peer reviewers first screen the outlines of proposals, and, in the second step, evaluate the full applications.

## The introduction of peer review

Expert assessment was common practice as early as in the 17^th^ century when scientific discoveries were discussed by scholars at meetings of the Royal Society in the UK. Peer review in the modern sense—that is, the organized search for expert's assessment on scientists’ discoveries—began with scientific publishing. The Royal Society's *Philosophical Transactions* is the first scientific journal that has used it to select manuscripts for publication.

The extensive use of peer review for distributing research funding coincided with the expansion of government funding of research in the USA and Europe after War World 2. Since peer review has become the main mechanism for distributing resources from governments and philanthropic funders, RAND Europe calculated that more than 95% of public funding for medical research in the UK was allocated by peer review each year. This year's budget for the largest funder of biomedical research in the world, the US National Institutes of Health (NIH), is US$39 billion; it awards more than 80% to more than 300,000 researchers using peer review (https://www.nih.gov/about-nih/what-we-do/budget). The US National Science Foundation (NSF) assigned more than 90% of its funding for research based on peer review in 2017 (https://www.nsf.gov/nsb/publications/2018/nsb201915.pdf), and the ERC distributed a 1.8 billion Euro budget using peer review in 2017 (https://erc.europa.eu/projects-figures/facts-and-figures). Wellcome Trust, one of the largest private funders in the world for biomedical research, distributed about GBP 640 million in grants in the financial year 2017/2018, using external and internal reviewers (https://wellcome.ac.uk/funding/people-and-projects/grant-funding-data/grant-data-2017-18).

Nonetheless, some of the weaknesses of peer review were already apparent at its wide adoption after 1945, and the challenges for peer review have grown drastically 70 years after it became the standard for evaluating research. The growing number of researchers competing for grants and positions, the growing number of scientific journals, the changes brought by the Internet, and more attention by politicians and the public to the results and conduct of research have all made the problems become more apparent.

The extensive use of peer review for distributing research funding coincided with the expansion of government funding of research in the USA and Europe after War World 2.

## Problems and weaknesses

The first of these problems is increasing reviewer fatigue. As peer review is ubiquitous and central to research, all researchers at a certain stage of their career become involved. Most scientists consider it a duty and invest a significant amount of their time without any compensation as review work is generally not recognized for hiring or promotion. A 2009 survey of 73 institutions in the USA found that faculty devoted 8% of their time to professional services, which includes grant and manuscript reviews 1.

In 2015, the NSF engaged 16,255 scientists to evaluate 51,588 proposals and estimated that the total time spent by reviewers amounted on average to 360 person‐years with each reviewer spending about 3.9 h in writing one review, not counting the time spent participating in panels (https://www.nsf.gov/nsb/publications/2016/nsb201641.pdf). The ERC engages 375 panel members plus 2,000 external referees for each call. In 2017, they reviewed more than 8,000 applications across all ERC schemes. The same year, the German Research Foundation (DFG) requested 22,500 reviews from more than 14,900 reviewers. These numbers are likely to increase, as the number of scientists competing for grants and funding grows. Many journals already notice a “reviewer fatigue”: more than 40% of researchers worldwide decline review requests owing to lack of time (https://publons.com/community/gspr). As the burden on individual researchers is increasing, reviewers find it increasingly difficult to devote enough time for high‐quality reviews. According to a researcher “There is a limit to how much a reviewer can do, and at some point, the criteria for evaluating get blurred and confusing.” (personal communication).

A further concern about traditional peer review is its inherent conservatism that may inhibit creativity. As funding is usually limited compared to the number of requests, reviewers tend to “play it safe” and select proposals that have better chances to succeed, rather than risky and ambitious ideas. In addition, review panels tend to settle on a consensus decision, which increases the tendency to dismiss risky, out‐of‐the‐box proposals 2.

Most evidence is anecdotal, but there are famous examples of bold and revolutionary research projects that were turned down by peer review, such as the NIH's rejection of Craig Venter's proposal to apply his newly developed technique of whole‐genome shotgun sequencing to sequence the genome of the *Haemophilus influenzae* bacterium. Venter pursued the work with his private company and developed what has become the most widely used method for gene sequencing. Both NIH and NSF refused to fund the work of Leon Cooper, a Nobel Prize Laureate in Physics, on neural networks. His research, which resulted in a large number of publications, was eventually funded by the Office of Naval Research (ONR), which uses a different method of evaluating funding proposals.

In addition, various criteria used by peer reviewers to allocating funding—notably the numbers of publications, citations and the name or impact factor of the journals where scientists publish their research—are being criticized for limiting creativity and the exploration of untested ideas. Such factors are believed to encourage applicants to publish as many papers as possible in one field, and even cut corners and exaggerate the significance of their results. Indeed, focusing on publications alone would quite likely have prevented creative thinkers such as Fred Sanger from getting funded. Sanger, who won two Nobel Prizes and revolutionized biological research, did not have many publications in high‐impact factor journals, and switched fields to tackle bold and untested ideas.

Another weakness of peer review is the potential influence of conscious or unconscious bias on reviewers’ decisions related to research field, affiliation, nationality, gender, age or ethnicity of the applicants. While there is no clear evidence, some funding agencies for instance found lower success rates for female scientists (https://bbsrc.ukri.org/documents/1511-understanding-app-rates-by-female-academics/), as well as lower success rates for non‐white applicants 3. The Swiss National Science Foundation (SNSF) found that reviewers nominated by applicants tended to score their applications higher than reviewers selected by the Foundation. It concluded that their peer‐review mechanism might be subject to bias and stopped requiring reviewer proposals by applicants [preprint: 4].

… the challenges for peer review have grown drastically 70 years after it became the standard for evaluating research.

These inherent weaknesses raise the question whether peer review is actually the best method for selecting the best people or projects. Some have tried to answer this by looking at the results of projects and researchers but the criteria have mainly been based on publication metrics: the number of papers produced, the numbers of papers published in high‐impact journals and the number of citations of those papers. Some studies found a positive correlation between reviewer's scores and bibliometric measures of funded projects, but a number of other analyses found only weak or no association. Similar comparisons based on career progressions of successful and unsuccessful applicants have found no significant difference either [preprint: 5]. It again points to the problem of using bibliometrics as main criteria to evaluate applicants, despite the advice of the San Francisco Declaration on Research Assessment (http://www.sfdora.org). If metrics were the main selection criteria, one could argue, why would we need peer review at all?

Another problem with peer review is that the aim of the selection process is sometimes not clear to either the funders or reviewers. The question whether peer review is good at selecting the best people or proposals is linked to the goals of the funding scheme. Is the aim to identify future success, to reward past success or to achieve specific scientific or social goals? Is it to select researchers who publish more papers or more high‐impact factor papers, or to select future leaders, or scientists who can accomplish the proposed research projects?

## Radical solutions

Given the problems of peer review, some have proposed to eliminate it altogether. One alternative would be distributing the available funding equally to all qualified researchers without any selection, so everyone would receive the same amount of money 6. It would save the time spent by scientists on assessing and writing applications and eliminate bias. An analysis of the amount of public funding that researchers would receive over a period of 5 years in the UK, the Netherlands and the USA suggests that equalitarian distribution of funds would give each scientist enough money for research and travel costs 7, but not enough to support large and expensive research projects.

… focusing on publications alone would quite likely have prevented creative thinkers such as Fred Sanger from getting funded.

Another way to select applications is relying on expert administrators to directly select proposals for funding, rather than seeking advice from external experts. This method is used by the US Defense Advanced Research Projects Agency (DARPA), which employs 100 qualified programme managers to distribute about US$3 million every year (https://www.darpa.mil/about-us/budget). The managers come from academia or industry, serve for a maximum of 4 years and are personally responsible for the success or failure of the funded project. They are in close contact with researchers to monitor progress and can stop projects that are not performing as expected. The decision‐making process is fast and empowers the managers to fund risky and unconventional research projects. DARPA had an important role, for example, in the development of computer technologies that preceded the Internet, and in biology, when it funded Eckard Wimmer's *de novo* synthesis of the polio virus in 2002 that paved the way for whole‐genome synthesis.

Other agencies use variants of this model. The US National Science Foundation employs managers from academia to choose projects for their Small Grant Scheme for Exploratory Research (https://www.nsf.gov/pubs/2005/nsf05053/nsf05053.jsp). The Wellcome Trust (WT) launched the *Leap Fund* in 2018 to fund high‐risk research projects in the health and life sciences. It is led by a CEO who decides which ideas to fund, which level of ambition and risk to allow, and who can reallocate funds as he/she sees appropriate (https://wellcome.ac.uk/press-release/wellcome-launches-%C2%A3250m-leap-fund-place-big-bets-bold-research). Applying this method to other funding schemes with smaller budgets might be difficult, but it will be interesting to see the outcomes of the WT scheme, and to compare it with traditional peer review.

## Incremental changes

Other changes have been suggested and implemented to address the weaknesses of traditional peer review. Some funding agencies ask peer reviewers to focus only on applicants’ past performance, rather than judging the validity of the proposed projects, with the idea that past success is the best predictor of future performance. The MacArthur Fellows Programme in the USA uses this method for funding projects in all areas (https://www.macfound.org/programs/fellows/strategy/). This method can also help to reduce conservatism, because scientists wanting to change fields or applicants with unconventional ideas can be funded on the basis of their past performance.

This is the stated goal of the Investigator Programme of the Howard Hughes Medical Institutes (HHMI), a large US non‐profit organization who “fund people, not projects” (https://www.hhmi.org/scientists) to carry out basic biomedical research. HHMI evaluates the overall quality of the science carried out by the applicants rather than the proposed projects. Moreover, it explicitly encourages researchers to take risks, explore unknown fields, and take failure into account. This comes with long‐term funding for seven or more years for “HHMI investigators” and has yielded 29 Nobel Prizes. A study comparing the careers of HHMI investigators and NIH investigators found that the former produce more high‐impact papers at a much higher rate than NIH scientists, and suggests that the HHMI funding scheme is effective in stimulating researchers to explore new research areas 8.

Other philanthropies have adopted different methods to spend their funds efficiently. The Bill & Melinda Gates Foundation (BMGF) is the largest private foundation with assets of about US$46.8 billion. The BMGF has formulated clear and practical goals and uses a straight‐forward decision‐making mechanism to achieve these. Rather than waiting for applicants to propose ideas and projects, it proactively identifies suitable applicants and solicits them to apply for funding. Internal programme officers are responsible for monitoring the performance of the funded project and work with the grantees to achieve the foreseen goals (https://docs.gatesfoundation.org/documents/our-approach-to-grants.pdf). The BMGF's funded research programmes have contributed, to, among others, innovative research on HIV, tuberculosis, malaria, the eradication of polio, improved water quality and sanitation and research on neglected tropical diseases.

Given the problems of peer review, some have proposed to eliminate it altogether.

The NSF is one of the few public funding agencies that specifically selects projects not only based on the intellectual merit of the proposals, but also on its potential broader impacts, such as the promotion of teaching and learning, the inclusion of under‐represented groups, and other benefits to society (https://www.nsf.gov/pubs/2007/nsf07046/nsf07046.jsp).

## Anonymous review and clear criteria

Anonymizing applications could help to reduce bias and encourage scientists to apply with unconventional and out‐of‐the‐box ideas. This is the idea behind the *The Villum Experiment*, a new funding programme launched in 2018 by the Villum and Velux Foundations in Denmark (https://veluxfoundations.dk/en/technical-and-scientific-research/villum-experiment). Indeed, an analysis of the effect of blinding applications in the first stage and disclosing identities in the second stage of the assessment of grant proposals in Spain found that reviewers did change their evaluation once they could see applicants’ identities 9; the authors of the study suggest that blinding application might help to limit reviewers’ conscious or unconscious bias.

Other measures to prevent or limit bias that have been implemented by funding agencies include training reviewers, publishing clear conflict of interest policies, providing reviewers with clear guidelines on the evaluation criteria, and making them aware of potential biases. The MRC in the UK has published online guidelines including assessment criteria and advice on how to control bias (https://www.mrc.ac.uk/documents/pdf/reviewers-handbook/). An evaluation of a training video for grant reviewers in the USA found that explicitly explaining the definitions and meaning of the rating scales used by the NIH and drawing reviewers’ attention on the consequences of their scorings, improved rating scale interpretation and application, both for experienced and unexperienced reviewers. The authors suggest that this highlights the importance of defining clear criteria and making reviewers aware of what is expected from them and why 10.

## Partial randomization

One proposal that has gained some popularity in recent years is the use of partial randomization, which complements traditional peer review with a lottery for a limited selection of applications. After selecting the top applications to be funded and those that are not, the remaining applications are all equally good and would be funded if there were enough money. Reviewers are faced with the difficult task of select among these, which increases the risk of arbitrary decisions that are not based on scientific criteria. It also increases the risk of bias. A draw would provide an unbiased measure of fairness and might limit applicants’ expectations on the objectivity of the peer‐review process.

A draw would therefore provide an unbiased measure of fairness and might limit applicants’ expectations on the objectivity of the peer review process.

So far, only a couple of funding agencies have been experimenting with partial randomization, such as the New Zealand Health Research Council with its Explorer Grants. Started in 2013, the scheme assigns NZ$ 150,000 a year to four innovative and out‐of‐the‐box research projects in all areas. All eligible proposals are considered equally deserving and are selected by a random process. In Europe, the Volkswagen Foundation has been testing partial randomization in its *Experiment! In Search of Bold Research Ideas* grants since 2017. The process selects 30–50 projects among 500 applications per year, each of which receives Euro 120,000 for 18 months. In the pilot phase that will run until 2020, the middle‐range proposals are divided into two groups, one of which is selected by peer review and the other by a lottery; the winners do not know how they were selected. The scheme is accompanied by a research project on its effects, and the results of the evaluation will be made public at the end of the pilot phase.

A major concern about partial randomization is that it is seen as contradicting traditional decision‐making based on merit. Researchers also fear to be stigmatized if they are selected by a lottery, and funders fear that it would impact negatively on the reputation of their schemes. Moreover, the use of a lottery, even a partial one, might give the impression to governments and the public that scientists cannot make decisions. However, at least younger researchers do not seem to be overly concerned: 66% of participants in an online survey by the EMBO Fellowship Programme would be in favour of partial randomization.

Another idea that has gained increasing interest is involving non‐experts into the review process: patients and their families, care takers and even the general public. The rationale behind patient engagement in biomedical research is obvious: the knowledge and personal experience of patients and their families can help to increase the quality and the validity of the research, and contributes to achieving the ultimate goal of improving patient care or developing new therapies. The Patient‐Centred Outcomes Research Institute (PCORI), an independent, non‐governmental organization, was the first major US funding agency to involve lay persons, patients, patient families and care givers in peer review in 2012. The Rare Disease Foundation in Canada involves patients and parents in the review process of their Microgrants for research into rare diseases to evaluate the potential impact on care, and the priorities of patients and their families (http://apps.rarediseasefoundation.com/microgrant/).

… as the public are the main funder of research, it is fair to involve representatives in the process of deciding how the money will be spent

An even more radical version is involving the public at large, together with scientific experts, in evaluating research proposals. The rationale here is that a wider range of knowledge and experience improves transparency, limits the danger of bias and contributes to addressing social benefits. Moreover, as the public are the main funder of research, it is fair to involve representatives in the process of deciding how the money will be spent. Health Research Board in Ireland, the largest funder for health‐related research in the country, started a pilot public review scheme for its Investigator‐Led Projects in 2017 and recruited participants via advertisements in the radio and other channels. During the first step of the project, public reviewers provided feedback directly to the applicants, and they will be involved in the expert panels that make the final funding decisions. The initial evaluation of the project was positive: researchers said that the public reviewers did have useful suggestions for their proposals and most of them would revise their applications accordingly (https://www.hrb.ie/funding/funding-schemes/public-and-patient-involvement-in-research/).

None of these changes will likely fix the weaknesses and problems of peer review. However, funders have the responsibility to ensure that their money is being distributed in the most efficient and fairest way and should not shy away from experimenting with new ideas to achieve this goal. Philanthropies and private funders have more freedom to explore alternatives, and governmental funders could learn from them. One potentially easy problem to fix would be deciding and communicating clearly the goals of a particular funding scheme to help reviewers select the most appropriate researchers and projects. The solution is that there is no one solution, but a variety of approaches and schemes can be used to ensure that the investments in scientific research meet the expectations of and are beneficial for society. Comparisons between different approaches should be done systematically and shared among funders. The collaboration with experts from the social sciences who study research assessment processes will be an essential part of this review process.

## Conflict of interest

Sandra Bendiscioli is a Policy Officer at EMBO, which is independent of EMBO Press.

**Further reading**

**On general problems with peer review**

1. Gropp RE, Glisson S, Gallo S, Thompson L (2017) Peer review: a system under stress. *BioScience* 67: 5
2. Gurwitz D, Milanesi E, Koenig T (2014) Grant application review: the case of transparency. *PLoS Biol* 12: e1002010
3. Gluckmann P (2012) Which science to fund: Time to review peer‐review? New Zealand Office of the Prime Minister's science advisory committee. https://www.pmcsa.org.nz/wp-content/uploads/Which-science-to-fund-time-to-review-peer-review.pdf
4. Ismail S, Farrands A, Wooding S (2009) *Evaluating Grant Peer Review in the Health Sciences. A review of the literature*. Santa Monica, CA: RAND Corporation
5. Guthrie S, Ghiga I, Wooding S (2018) *What do we know about grant peer‐review in the health sciences?* Santa Monica, CA: RAND Corporation

**On bias in peer review**

1. Kaatz A, Lee YG, Potvien A, Magua W, Filut A, Bhattacharya A, Leatherberry R, Zhu X, Carnes M (2016) Analysis of National Institutes of Health R01 application critiques, impact and criteria scores: does the sex of the Principal Investigator make a difference? *Acad Med* 91: 1080–1088
2. Frith U (2017) Fighting bias with quotas and lottery, Social Minds blog. http://frithmind.org/socialminds/2017/07/28/fighting-bias-with-quota-and-lottery/

**On conservatism of peer review**

1. Alberts B, Kirschner MW, Tilghman S, Varmus H (2014) Rescuing US biomedical research from its systemic flaws. *Proc Natl Acad Sci USA* 111: 5773–5777
2. Brenner S (2014) Frederick Sanger (1918–2013). *Science* 343: 262
3. CookDeegan RM (1997) Does NIH need a DARPA? Issues in Science and Technology 13: 25–28

**On the effectiveness of peer review**

1. Fang FC, Bowen A, Casadevall A (2016) NIH peer review percentile scores are poorly predictive of grant productivity. *eLife* 5: e13323
2. Lindner MD, Vancea A, Chen M‐C, Chacko G (2016) NIH Peer Review – Scored review criteria and overall impact. *Am J Eval* 37: 238–249
3. Li D, Agha L (2015) Big names or big ideas: do peer‐review panels select the best science proposals? *Science* 348: 434–438
4. Bornmann L, Wallon G, Ledin A (2008) Does the committee peer‐review select the best applicants for funding? An investigation for the selection process for two European Molecular Biology Organization programmes. *PLoS One* 3: e3480

**On lotteries**

1. Fang FC, Casadevall A (2016) Research funding: the case for a modified lottery. *mBio* 7: e00422–16
2. Stone P (2009) The logic of random selection. *Polit Theory* 37: 375–397
